# Coping Using Sex, Health-Related Behaviors, and Mental Health During COVID-19 Lockdown in the UK

**DOI:** 10.3389/fpsyt.2022.880454

**Published:** 2022-05-24

**Authors:** Natasha Daly, Andrew Jones, Carlo Garofalo, Kasia Uzieblo, Eric Robinson, Steven M. Gillespie

**Affiliations:** ^1^Department of Primary Care and Mental Health, University of Liverpool, Liverpool, United Kingdom; ^2^Department of Psychological Sciences, University of Liverpool, Liverpool, United Kingdom; ^3^Department of Developmental Psychology, Tilburg University, Tilburg, Netherlands; ^4^Forensic Care Specialists, Utrecht, Netherlands; ^5^Criminology Department, Vrije Universiteit Brussel, Brussel, Belgium

**Keywords:** COVID-19, health behavior, coping, sex, depression, anxiety

## Abstract

**Background:**

Peoples' sexual behaviors have changed during the period of enforced COVID-19 social distancing, in some cases, to cope with negative feelings during lockdown. Research on coping using sex is relatively restricted to samples of men with a history of sexual offending, and it is unknown whether coping using sex is associated with health-related behaviors and mental health in the general population.

**Aim:**

We examined if coping using sex before and during lockdown was associated with adverse outcomes (i.e., self-perceived reduction in health-related behaviors and mental health) in a community sample.

**Hypotheses:**

We hypothesized that participants who reported greater use of sex to cope in the weeks preceding lockdown would show a greater decline in health-related behaviors and mental health during lockdown. Furthermore, that changes in coping using sex resulting from lockdown would account for further variance in the worsening of health-related behaviors and mental health.

**Methods:**

Participants were UK residents, aged 18–60 years, and fluent in English. 789 participants completed an online survey, providing demographic information, self-reported social distancing, loneliness, and coping using sex over a 14-day period during lockdown, and retrospectively preceding lockdown.

**Outcomes:**

Participants reported perceived changes in health-related behaviors and mental health symptomatology during lockdown compared to before the pandemic. They also self-reported levels of stress, anxiety and depression during lockdown.

**Results:**

Greater coping using sex prior to lockdown predicted positive change in health-related behaviors, for example, higher scores were associated with participants reporting having exercised and slept more. It was also associated with higher trait levels of anxiety, stress and depression during lockdown. Changes in coping using sex from before to during lockdown did not predict perceived changes in health related behaviors or mental health symptomatology.

**Conclusions:**

Overall, greater coping using sex prior to lockdown was associated with worse mental health symptomatology during lockdown (anxiety, depression and stress), however, it was also associated with perceived positive change in health-related behaviors compared with before lockdown. This suggests that coping using sex may be associated with negative emotional reactions during lockdown, but may also be linked with positive change in health-promoting behaviors.

## Introduction

The coronavirus disease 2019 (COVID-19) outbreak has presented a variety of economic, psychosocial and health-related stressors that are likely to have a considerable impact on mental health and well-being for some individuals (1, 2). The rapid spread of COVID-19 led to the introduction of social distancing measures in many countries worldwide to reduce infection rates. However, these measures have also been found to have had short- and long-term adverse consequences on mental health and well-being (3). Indeed, longitudinal studies indicate heightened prevalence of mental health problems in the UK during April, 2020 (one month into a national “lock-down”) compared with 2017–2019 (4). This heightened prevalence remained evident in May and June, 2020 (4). There is also evidence that health related behaviors were also impacted. For example, UK adults reported negative changes in eating behaviors and physical activity, and experienced more barriers to weight management compared to before lockdown (5). It is also expected that some people will use problematic coping behaviors, such as alcohol use (6) and unhealthy eating behaviors, (7) to cope with the stress of the pandemic (8).

Coping is defined as a person's cognitive and behavioral efforts to manage specific external and/or internal demands that are appraised as stressful or exceeding the person's resources (9). The Threat Appraisal and Coping Theory (9) suggests that both adaptive and maladaptive coping strategies should be considered as a response to cognitive appraisals of a stressful situation or condition. While both adaptive and maladaptive coping serve the function of attempting to reduce the perceived stress associated with the triggering condition, they differ in the outcome they elicit. Adaptive coping involves behaviors that are linked with positive outcomes; for example, exercise, good sleep hygiene, and social support seeking are forms of adaptive coping (10–15). In contrast, maladaptive coping behaviors are associated with negative outcomes; for example, binge eating, self-injurious behavior, and problem gambling (16, 17). In short, the evaluation of a coping strategy is tied to its adaptive or maladaptive effect on one's physical and mental well-being in the short and/or long term.

While the adverse psychological effects of the COVID-19 lockdown have been extensively studied in community samples [see (18) for a review and meta-analysis], the investigation of potential maladaptive strategies used to cope with the COVID-19 pandemic has received relatively little attention. Several studies have examined the use of coping mechanisms including increased social support (19), not reading news related to COVID-19 too often, pursuing hobbies and going outdoors (20), to manage the impact of lockdowns and social restrictions on psychological health, stress and loneliness. Relatively fewer studies have examined the use of maladaptive coping strategies, although increases in high-risk drinking patterns and unhealthy eating behaviors have been reported (7).

A further strategy used by some people to cope with negative or adverse events is engaging in sexual behaviors (21). There is accumulating evidence that people's sexual behaviors changed during the period of social distancing due to COVID-19, including increased Pornhub traffic (22), greater use of sex toys during masturbation (23), and expanding one's sexual repertoire (24). There is also evidence to suggest that whilst solitary sexual behaviors (e.g., masturbation and porn usage) have increased, the frequency of sexual intercourse has decreased (25).

There is some evidence to suggest that using sex to cope with stressful and problematic situations can be maladaptive for some people. For example, some people who have sexually offended use sex to cope with negative emotions and this becomes part of their offense pathway (26–28). Despite these reports, only one study to our knowledge has examined the correlates associated with coping using sex in non-forensic samples (21), and no studies have examined the effect of changes in coping using sex in response to a stressor such as the current pandemic and associated lockdown. In the context of the COVID-19 pandemic, coping using sex may represent a largely adaptive function of spending more time at home with a romantic or sexual partner, especially during social lockdown when usual rewarding activities are not permissible. In light of the evidence that peoples sexual behaviors have changed during the COVID-19 pandemic, it is especially important to understand the potential health-related behaviors, and mental health outcomes, associated with coping using sex.

One previous study examined the correlates of using sex to cope during the initial period of national lockdown to prevent the spread of COVID-19 in the UK. In this study, participants were asked to self-report their use of sex as a coping strategy for managing negative situations. The measure of coping using sex included items that enquired about the use of consensual sexual behaviors, but also non-consensual behaviors including rape and child abuse. Even though no significant changes were found in mean levels of coping using sex before vs. during the lockdown, coping using sex was associated with several correlates suggestive of negative psychological adjustment, including emotion regulation difficulties, less adherence to social distancing, and loneliness in men (21). Greater difficulties in emotion regulation were also associated with a greater likelihood of endorsing items related to coping with a theme of rape/violence for women but not men (21).

The present study sought to extend this work by examining the outcomes associated with coping using consensual sex during lockdown, including health-related behaviors, and mental health symptomatology, in a community sample in the UK. Based on previous findings of the largely maladaptive correlates of coping using sex, we hypothesized that participants who reported greater use of sex to cope in the weeks preceding lockdown would show an overall greater self-perceived worsening of both health-related behaviors (e.g., binge eating, drinking alcohol) and mental health symptomatology (e.g., felt anxious, felt depressed) during lockdown. We also hypothesized that a relative increase in coping using sex during lockdown (compared with a two-week period preceding lockdown) would account for further variance in the change of health-related behaviors and mental health symptomatology.

While these hypotheses were pre-registered (see below for details) we further examined in an exploratory fashion whether coping using sex was associated with absolute levels (as opposed to changes) in mental health outcomes, as well as the possible moderating role of gender in each of the associations investigated. As we previously identified an association between coping using sex and emotion regulation difficulties,[Gillespie et al. (21)] we hypothesized that greater coping using sex prior to lockdown would be associated with higher trait levels of mental health symptomology (i.e., depression, anxiety and stress) during lockdown.

## Methods

### Data

The data used in this study represents a subset of the data collected by Gillespie et al. (21) and Robinson et al. (29). The data were collected through an online cross-sectional survey, designed and presented to participants using Inquisit Lab. In the present study, we used data previously reported by Gillespie et al., who examined the effects of social distancing, loneliness, difficulties in emotion regulation, and self-regulation on participants self-reported coping using sex during lockdown in the United Kingdom. In the current study, we aim to investigate whether coping using sex before and during the COVID-19 lockdown was associated with adverse outcomes (e.g., self-perceived reduction in health-related behaviors and mental health). The outcome variables examined in the current paper were not used in the previous study by Gillespie et al., but instead represent a subset of variables gathered by Robinson, et al. The aims hypotheses and analyses reported here have not previously been tested in either of these reports.

### Participants

Social distancing and lockdown were officially ordered by the U.K. Government on March 23, 2020. All participants were recruited via Prolific Academic between April 19, 2020 and April 21, 2020, a period during which there was closure of non-essential shops and “stay at home” orders across the UK. Prolific Academic provides a platform for crowdsourcing participants into research studies (30). Participants recruited via Prolific Academic have been found to produce high quality data from a more diverse population than similar recruiting tools (e.g., MTurk, Crowd-Flower) (30). Participants were paid in line with Prolific Academic payment guidelines at a rate of £6.74 per hour, resulting in a final payment of £1.68 for the task which lasted approximately 15 min.

In order to participate, individuals had to be aged between 18 and 60 years, a resident of the United Kingdom, speak fluent English, and have an Internet connection. Participants were instructed not to participate if they had consumed alcohol in the previous 24 h or if sensitive questions about sexual behavior, or health-related behaviors and mental health were likely to cause distress. Alcohol consumption was not explicitly controlled for but relied on the participants' word; participants were asked to check a box on the consent form stating “I confirm that I have not/will not consume alcohol on the day of completing the measures as this may affect my responses to the task.”

### Measures

#### Demographics

Participants completed demographic measures of gender, age, ethnicity, highest education level, living status, previous diagnosis of psychiatric condition, self-reported height and weight. Next, participants completed items on whether they had been formally diagnosed with or suspected they previously/currently had COVID-19 and indicated if they had any of 10 medical conditions (e.g., diabetes, weakened immune system, BMI ≥40 kg/m^2^) identified by the UK NHS (31) as placing them at “high risk” for COVID-19. The survey also included attention check questions to identify any careless responders.

We created binary variables for ethnicity (white—other), psychiatric diagnosis (present—absent), education (educated to degree level and above—not educated to degree level), being in a high-risk group for difficulties resulting from COVID-19 (present—absent), and living status (alone—with others). We calculated participants' BMI by dividing their self-reported weight in kilograms by their self-reported height in meters squared.

#### Social Distancing

We modified a social distancing questionnaire that was designed in the context of COVID-19 to assess the extent to which participants were observing social distancing advice (32). Participants were asked in the last 2 weeks how much time they had spent with friends, immediate family, colleagues, and usual social network in person, with the anchors 1 (*not at all*) to 5 (*very often*). The original scale, which was designed for use with adolescents, was modified for use with adults by replacing an item that asked about “time spent with others (e.g., teachers or neighbors)” with an item that asked about “time spent with others (e.g., colleagues or neighbors).” Items were reverse scored so greater scores indicated increased social distancing. Participants were also asked about their social media use to connect/play games with friends and family, individuals/groups outside of their usual contacts using the same anchors. Both scales showed good internal consistency (physical social distancing *N* = 789; ω = 0.638; social media use *N* = 789; ω = 0.735). Only items asking about physical social distancing were included in the current analyses.

#### UCLA Loneliness Scale

The UCLA loneliness scale (33) is a 20-item Likert scale which measures subjective feelings of loneliness and social isolation (e.g., “I lack companionship”), with the anchors “*I often feel this way*,” “*I sometimes feel this way*,” “*I rarely feel this way*,” “*I never feel this way*.” Participants were asked to think about the “last 2 weeks” when responding. A total score for loneliness is calculated by summing the scores from each question. The scale has good psychometric properties (33). The internal consistency in this data set was excellent (*N* = 789; ω = 0.952).

#### Coping Using Sex Inventory

Participants completed the 16-item CUSI, (26) which provides a series of scenarios and asks participants to indicate using a Likert scale from 1 (*not at all*) to 5 (*very often*) how often they engaged in these behaviors when encountering a difficult, stressful or upsetting situation. This measure has previously been used to measure coping using sex in non-forensic samples (34). The inventory consists of three subscales, asking about sexual thoughts and behaviors with themes of consent, rape, and child sexual abuse. The scale also yields an overall score, with higher scores indicating more frequent use of sex to cope. An example of a consensual item is “*I have fantasized about having sex with a consenting adult*,” while an example of a rape item is “*I have forced my regular partner to have sex*.” In a single session, participants were asked to complete this scale corresponding to two separate time periods: (i) in the two weeks before lockdown was introduced by the U.K. government, and (ii) in the last two weeks. Thus, reported levels of coping using sex before lockdown were based on retrospective reports. The scale had good internal consistency for both scenarios (before lockdown *N* = 760; ω = 0.803; two previous weeks ω = 0.698).

Both the rape subscale and child sexual abuse subscale were highly positively skewed toward zero; for the child sexual abuse subscale only 1% of participants endorsed at least one of the items. We therefore examined responses to the six consent subscale items only, excluding child (*n* = 4) and rape (*n* = 6) items. These consensual items included themes of fantasies, masturbation, pornography use, and sexual acts involving consenting adults. Although data were collected using the full scale, in our analyses we do not include any of the CUSI items that enquire about abusive or illegal sexual behaviors. For the analyses reported here, we used consent subscale scores prior to lockdown and change in consent subscale scores from pre- to post-lockdown.

#### Depression, Anxiety, and Stress Scale (DASS-21)

Participants completed the 21-item DASS, (35) which provides a list of statements and asks participants to indicate on a four-point response scale from 0 (did not apply to me at all) to 3 (*applied to me very much or most of the time*) how often each statement applied to them over the last week. The scale consists of three subscales, asking about feelings of depression, anxiety, and stress, with higher scores indicating greater mental health symptomatology. An example of a depression item is “*I felt down-hearted and blue*”, an anxiety item is “*I found myself getting agitated*”, while an example of a stress item is “*I found it difficult to relax*”. The scale had good internal consistency (stress *N* = 765, ω = 0.885; anxiety ω = 0.797; depression ω = 0.909).

#### Perceived Changes Since COVID-19

The Perceived changes since COVID-19 scale (29) was developed for use in the current study and has previously been reported on by Gillespie et al., (21) and Robinson et al., (29) in the same data set. Participants were asked to report changes in 15 behaviors related to physical health, mental health, and interpersonal functioning compared with before the COVID-19 pandemic. Data were used from six items that enquired about changes in health-related behaviors, and four items that enquired about changes in mental health, as follows: “Compared with before the COVID-19 virus crisis, I have exercised/ slept/ eaten healthily/ binged on food/ drank alcohol/ smoked/ felt depressed/ felt anxious/ intentionally harmed myself/ had suicidal thoughts.” Participants were asked to respond to each item on the same seven-point Likert scale (1 = *a lot less than usual*, 2 = *less than usual*, 3 = *a little less than usual*, 4 = *about the same*, 5 = *a little more than usual*, 6 = *more than usual*, and 7 = *a lot more than usual*).

##### Health Related Behaviors

To calculate participants' perceived change in health related behaviors during COVID-19, we summed scores across the six health-related behavior items included in the “Perceived changes since COVID-19” questionnaire: “I have exercised”, “I have slept”, “I have eaten healthily”, “I have binged on food”, “I have drank alcohol” and “I have smoked”. Reverse scoring was employed for the items: binged on food, drank alcohol and smoked, so that higher scores indicated an increase in healthy behaviors. We also created a count variable that indicated the number of health-related behaviors for which participants reported a worsening, whereby higher scores indicated a deterioration in a higher number of health-related behaviors. Although the scale had low internal consistency (N = 727; ω = 0.514), this value is difficult to interpret for a small scale (6 items).

##### Mental Health

To calculate participants' perceived change in mental health during COVID-19, we summed scores across the four mental health items included in the “Perceived changes since COVID-19” questionnaire: “I have felt depressed”, “I have felt anxious”, “I have intentionally harmed myself”, and “I have had suicidal thoughts”. Higher scores indicated a perceived worsening in mental health. We also created a count variable that indicated the number of mental health items for which participants reported a worsening, whereby higher scores indicated a deterioration in a higher number of mental health items. The scale had acceptable internal consistency (*N* = 727; ω = 0.680).

### Procedure

Upon signing up to the study, participants were first presented with a landing page discussing the sensitive nature of some of the questionnaires. Prior to providing consent, participants were informed that the study covered sensitive questions relating to both legal and illegal sexual behaviors, health-related behaviors and mental health, and that they should not take part if such topics were likely to cause distress. The participant consent form included a point stating the following: “I understand that taking part in the study will involve completing a speeded response task and some questionnaires that enquire about social distancing and sexual behaviors, feelings of anxiety/sadness, and intentional harm/suicidal thoughts. I understand that some items will ask about my engagement in criminal acts and that these questions may cause distress”.

Participants then completed the demographic items and self-report measures; self-report measures were presented to participants in a random order. Two attention check questions (e.g. “*What planet do you live on?”)* were included to identify and remove careless responders. The survey also included additional items and a response inhibition task that are reported in separate manuscripts (21, 29). The study took approximately 15 min to complete, and participants received a small monetary reimbursement. Following completion of the study, participants were also shown a debrief form with signposting to a range of organizations offering support relevant to the topics covered in this study.” and then asked to read the information sheet and provide consent.

### Analysis Plan

The main analyses were preregistered on the Open Science Framework (OSF; osf.io/n24qj) together with all measures. We pre-registered our analysis plan following data collection but prior to examining data for the present research study. Analyses were conducted using jamovi v1.2 (36) running in the R environment (36). We used multiple linear regressions to test the effects of coping using consensual sex before lockdown and changes in coping using consensual sex compared to pre-lockdown on overall change in health-related behaviors and mental health separately. To investigate the effects of coping using sex before lockdown and changes in coping using sex on the number of health-related behaviors showing a worsening, we used ordinal regression. We focused analyses on perceived change in mental health on items measuring anxiety and depression (see Supplementary Material 2 for more information).

For each regression analysis, in the first model we entered gender, age, ethnicity (white vs. not), highest education level (educated to degree level and above vs. not educated to degree level), living status (alone vs. with others), previous diagnosis of psychiatric condition (yes vs. no), BMI, and COVID-19 high-risk health group (yes vs. no) as predictor variables and perceived change in health-related behavior and mental health as the outcome variables. In the second model, we included loneliness and physical social distancing as predictors. In the third model, we included pre-lockdown CUSI score and in the final model, we included change in CUSI (CUSI score during lockdown—CUSI score pre-lockdown) as a predictor.

In order to understand how coping using sex was associated with absolute levels of depression, anxiety, and stress during lockdown (i.e., not change scores), we performed unregistered exploratory linear regressions (as described above) using DASS-21 subscales as outcome variables (35). The results of these exploratory analyses are shown in Supplementary Material 3. We also conducted exploratory analyses to understand how the relationships between coping using sex prior to lockdown and each of the outcome variables described above might differ based on gender. We included the interaction of coping using sex prior to lockdown with gender as a predictor in the final step of each model, to replace change in coping using sex.

## Results

### Sample

Of the 907 individuals who accessed the survey, responses from 109 individuals who failed one or both manipulation checks, and nine duplicate responses were removed. Of the remaining 789 participants, we excluded any participants with missing data for variables used in this study. As we were using BMI as a predictor variable, we also excluded participants with implausible weight: <30–>250 kg, or height values: <120–>3 m (37, 38). Data from a total of 727 participants were used in the final analysis. See Table 1 for sample characteristics and Supplementary Table S1 in Supplementary Material 1 for the zero order correlations between predictor and outcome variables.

**Table 1:** Descriptive statistics (*N* = 727).

	**Min**.	**Max**.	***N*** **(%)/Mean (SD)**	**Median**
Gender—male	-	-	239 (32.9%)	-
Age	18	59	30.55 (9.64)	29
Ethnicity—white	-	-	581 (79.9%)	-
Highest education level—degree and above	-	-	471 (64.8%)	-
Living status—alone	-	-	71 (9.8%)	-
Psychiatric condition—yes	-	-	229 (31.5%)	-
BMI	14.52	53.50	25.84 (6.16)	24.16
COVID-19 high risk group—yes	-	-	137 (18.8%)	-
Loneliness	0	58	22.77 (13.68)	22
Social distancing (physical subscale)	1	5	4.68 (0.55)	5
Coping using sex prior to lockdown (consent subscale)	5	25	11.42 (4.71)	11
Coping using sex during lockdown (consent subscale)	5	25	11.42 (4.90)	10
Perceived change in health related behaviors (total)	9	42	25.03 (4.91)	25
Number of health related behaviors worsened	0	6	1.76 (1.37)	2
Perceived change in anxiety (item)	1	7	4.73 (1.32)	5
Perceived change in depression (item)	1	7	4.33 (1.30)	4
Depression subscale of DASS-21	0	21	6.52 (5.21)	5
Anxiety subscale of DASS-21	0	21	3.13 (3.42)	2
Stress subscale of DASS-21	0	21	6.35 (4.73)	6

*Descriptive statistics are reported only for participants that completed all measures reported in this study. For loneliness, social distancing, coping using sex prior to lockdown and coping using sex during lockdown, higher scores indicate increased levels*.

### Perceived Change in Health-Related Behaviors (Total Score)

The results of the linear regression analysis for perceived change in health-related behaviors (total score) are shown in Table 2. Model one [*F* (8, 718) = 4.47, *p* < 0.001] explained 4.7% of the variance in perceived change in health-related behaviors. Model two explained 5.9% of the total variance, representing a statistically significant improvement [Δ*R*^2^ = 0.011, *F* (2, 716) = 4.45, *p* = 0.015]. Model three explained 6.4% of the total variance in perceived change in health-related behaviors, again a statistically significant improvement [Δ*R*^2^ = 0.005, *F*(1, 715) = 4.17, *p* = 0.041]. The inclusion of change in coping using sex scores in model four did not significantly improve the model [Δ*R*^2^ < 0.001, *F* (1, 714) = 0.35, *p* = 0.558].

**Table 2:** Results of multiple linear regression on perceived change in health-related behaviors.

**Predictor**	**Estimate**	***SE***	***t***	***p***
**Model 3**
**Gender:**
Male—female	−0.674	0.430	−1.569	0.117
**Age**	−0.037	0.020	−1.805	0.071
**Ethnicity:**
White—not white	−1.447	0.460	−3.143	0.002**
**Education:**
Degree and above—lower than degree	−0.200	0.382	−0.523	0.601
**Living status:**
Alone—not alone	−1.088	0.602	−1.808	0.071
**Previous diagnosis of psychiatric condition:**
Yes—no	−0.908	0.413	−2.199	0.028**
**BMI**	−0.046	0.032	−1.464	0.144
**COVID-19 high risk health group:**
High risk condition—no high-risk condition	0.228	0.479	0.476	0.634
**Loneliness**	−0.027	0.014	−1.928	0.054
**Social distancing**	0.745	0.325	2.296	0.022*
**Coping using sex prior to lockdown**	0.088	0.043	2.042	0.041*

* *Indicates a significance level < 0.050*.

** *Indicates a significance level < 0.010*.

Based on the most parsimonious model (model 3), adhering to social distancing and greater levels of coping using sex prior to lockdown predicted more positive change in health-related behaviors. Being white and having a diagnosed psychiatric condition predicted a perceived worsening in health-related behaviors. For model 1 and model 2 parameters, see Supplementary Table S1 in Supplementary Material 2.

In an exploratory analysis, we replaced the addition of change in coping using sex in the final step, with the two-way interaction of gender with coping using sex prior to lockdown. This step did not significantly improve the overall model [Δ*R*^2^ < 0.001, *F* (1,714) = 0.011, *p* = 0.917].

### Number of Health-Related Behaviors Worsened

The results of the ordinal logistic regression analysis for the number of health-related behaviors for which participants reported a worsening are shown in Table 3. Model one [χ^2^(8) = 21.40, *p* = 0.005], explained 0.9% of the variance in the number of health-related behaviors worsened. Model two explained 1.6% of the total variance, a statistically significant improvement [${\Delta R}_{McF}^{2}$ = 0.005, χ^2^(2) = 16.03, *p* < 0.001]. The addition of coping using sex prior to lockdown [model 3; χ^2^(1) = 0.02, *p* = 0.884] and change in coping using sex [model 4; χ^2^(1) = 1.59, *p* = 0.208] did not significantly improve model fit.

**Table 3:** Results of ordinal logistic regression on no. health-related behaviors worsened.

						**95% Confidence interval**	
**Predictor**	**Estimate**	***SE***	***Z***	***p***	**Odds ratio**	**Lower**	**Upper**
**Model 2**
**Gender:**							
Male—female	−0.032	0.148	−0.218	0.827	0.968	0.724	1.294
**Age**	−0.005	0.007	−0.727	0.468	0.995	0.980	1.009
**Ethnicity:**							
White—not white	0.286	0.169	1.691	0.091	1.331	0.956	1.855
**Education:**							
Degree and above—lower than degree	−0.138	0.144	−0.956	0.339	0.871	0.656	1.156
**Living Status:**							
Alone—not alone	0.172	0.224	0.769	0.442	1.188	0.765	1.842
**Previous diagnosis of psychiatric condition:**							
Yes—no	0.219	0.155	1.414	0.157	1.245	0.919	1.689
**BMI**	0.020	0.012	1.703	0.088	1.020	0.997	1.044
**COVID-19 High Risk Health Group:**							
High risk condition—no high-risk condition	−0.106	0.179	−0.590	0.555	0.900	0.632	1.278
**Loneliness**	0.019	0.005	3.616	<0.001***	1.019	1.009	1.030
**Social distancing**	−0.167	0.127	−1.316	0.188	0.846	0.660	1.085

*** *Indicates a significance level < 0.001*.

Based on the most parsimonious model (model 2), greater loneliness predicted a worsening in a greater number of health-related behaviors. For model 1 parameters, see Supplementary Table S2 in Supplementary Material 2.

In an exploratory analysis, we replaced the addition of change in coping using sex in the final step, with the two-way interaction of gender with coping using sex prior to lockdown. This step did not significantly improve the overall model [${\Delta R}_{McF}^{2}$ =.016, χ^2^(1) = 0.047, *p* = 0.828]. In Supplementary Table S3 in Supplementary Material 2, we report the results of a Confirmatory Factor Analysis on the perceived change in health-related behavior scale. This analysis suggested that there was a single factor solution including five out of the six items, with the smoking item excluded. We therefore repeated the regression analysis using this new five-item scale. The results of this analysis are largely similar to the results reported here and are reported in full in Supplementary Tables S4, S5 in Supplementary Material 2.

### Perceived Change in Mental Health

#### Anxiety Item

The results of the multiple linear regression for anxiety are shown in Table 4. Model 1 [*F* (8, 718) = 6.76, *p* < 0.001] explained 7.0% of the variance in perceived change in anxiety. Model 2 explained 11.0% of the total variance, a statistically significant improvement [Δ*R*^2^ = 0.040, *F* (2,716) = 16.25, *p* < 0.001]. The addition of coping using sex prior to lockdown [model 3; Δ*R*^2^ =.002, *F* (1,715) = 1.81, *p* =.179] and change in coping using sex [model 4; Δ*R*^2^ = 0.002, *F* (1,714) = 1.61, *p* = 0.204] did not significantly improve model fit.

**Table 4:** Results of multiple linear regression on change in anxiety.

**Predictor**	**Estimate**	***SE***	***t***	***p***
**Model 2**
**Gender:**
Male—female	−0.525	0.103	−5.084	<0.001***
**Age**	−0.013	0.005	−2.527	0.012*
**Ethnicity:**
White—not white	0.138	0.120	1.154	0.249
**Education:**
Degree and above—lower than degree	0.108	0.100	1.079	0.281
**Living status:**
Alone—not alone	−0.118	0.158	−0.747	0.455
**Previous diagnosis of psychiatric condition:**
Yes—no	0.079	0.108	0.729	0.466
**BMI**	0.002	0.008	0.199	0.842
**COVID-19 high risk health group:**
High risk condition—no high-risk condition	0.080	0.126	0.640	0.522
**Loneliness**	0.020	0.004	5.588	<0.001***
**Social distancing**	−0.067	0.085	−0.792	0.429

* *Indicates a significance level < 0.050*.

*** *Indicates a significance level < 0.001*.

Based on the most parsimonious model (model 2), being male and being older predicted perceived reduction in anxiety levels, whilst being lonely predicted an increase in anxiety. For model 1 parameters, see Supplementary Table S1 in Supplementary Material 3.

In an exploratory analysis, we replaced the addition of change in coping using sex in the final step, with the two-way interaction of gender with coping using sex prior to lockdown. This step did not significantly improve the overall model [Δ*R*^2^ < 0.001, *F* (1,714) = 0.043, *p* = 0.836].

#### Depression Item

The results of the multiple linear regression for depression are shown in Table 5. Model 1 [*F* (8,718) = 7.48, *p* < 0.001] explained 7.7% of the variance in perceived change in depression. Model two explained 18.3% of the total variance, a statistically significant improvement [Δ*R*^2^ =.106, *F* (2,716) = 46.66, *p* < 0.001]. The addition of coping using sex prior to lockdown [model 3; Δ*R*^2^ = 0.002, *F* (1,715) = 1.39, *p* = 0.238] and change in coping using sex [model 4; Δ*R*^2^ < 0.001, *F* (1,714) = 0.14, *p* = 0.710] did not significantly improve model fit.

**Table 5:** Results of multiple linear regression on change in depression.

**Predictor**	**Estimate**	***SE***	***t***	***p***
**Model 2**
**Gender:**
Male—female	−0.293	0.098	−2.999	0.003**
**Age**	−0.013	0.005	−2.749	0.006**
**Ethnicity:**
White—not white	0.315	0.113	2.786	0.005**
**Education:**
Degree and above—lower than degree	0.032	0.095	0.344	0.731
**Living status:**
Alone—not alone	0.110	0.149	0.741	0.459
**Previous diagnosis of psychiatric condition:**
Yes—no	0.138	0.102	1.349	0.178
**BMI**	0.005	0.008	0.616	0.538
**COVID-19 high risk health group:**
High risk condition—no high-risk condition	−0.025	0.118	−0.213	0.832
**Loneliness**	0.033	0.003	9.660	<0.001***
**Social distancing**	0.046	0.080	0.570	0.569

** *Indicates a significance level < 0.010*.

*** *Indicates a significance level < 0.001*.

Based on the most parsimonious model (model 2), being male and being older predicted improvement in depression levels, whilst being white and being lonelier predicted an increase in depression. For model 1 parameters, see Supplementary Table S2 in Supplementary Material 3.

### Exploratory Analyses

#### DASS-21 Total Score

The results for all models of the linear regression analysis for depression during lockdown are shown in Table 6. Model one [*F* (8,717) = 19.53, *p* < 0.001] explained 17.9% of the variance in total DASS score during lockdown. Model two explained 41.8% of the total variance, a statistically significant improvement [Δ*R*^2^ =.240, *F* (2,715) = 51.44, *p* < 0.001]. Model three explained 42.8% of the total variance in total DASS score during lockdown, again a statistically significant improvement [Δ*R*^2^ = 0.009, *F* (1,714) = 48.51, *p* = 0.002]. Adding the change in coping using sex in model four did not significantly improve the overall model [Δ*R*^2^ < 0.001, *F* (1,713) = 1.44, *p* = 0.624].

**Table 6:** Results of multiple linear regression on DASS-21 total score during lockdown.

**Predictor**	**Estimate**	***SE***	***t***	***p***
**Model 3**
**Gender:**
Male—female	−3.185	0.821	−3.877	<0.001***
**Age**	−0.105	0.039	−2.708	0.007
**Ethnicity:**
White—not white	0.384	0.880	0.437	0.663
**Education:**
Degree and above—lower than degree	0.611	0.730	0.837	0.403
**Living status:**
Alone—not alone	−1.505	1.148	−1.310	0.191
**Previous diagnosis of psychiatric condition:**
Yes—no	5.103	0.788	6.476	<0.001***
**BMI**	0.033	0.060	0.547	0.585
**COVID-19 high risk health group:**
High risk condition—no high-risk condition	−0.505	0.914	−0.552	0.581
**Loneliness**	0.452	0.027	17.030	<0.001***
**Social distancing**	−0.049	0.619	−0.079	0.937
**Coping using sex prior to lockdown**	0.281	0.082	3.403	0.001***

*** *Indicates a significance level < 0.001*.

Based on the most parsimonious model (model 3), being male predicted lower total DASS score, whilst having a psychiatric condition, being more lonely and greater use of sex to cope prior to lockdown predicted greater total DASS score. For model 1 and 2 parameters, see Supplementary Table S6 in Supplementary Material 3.

In an exploratory analysis, we replaced the addition of change in coping using sex in the final step, with the 2-way interaction of gender with coping using sex prior to lockdown. This step did not significantly improve the overall model [Δ*R*^2^ <0.002, *F* (1,713) = 2.383, *p* = 0.123].

In an exploratory analysis, we replaced the addition of change in coping using sex in the final step, with the 2-way interaction of gender with coping using sex prior to lockdown. This step did not significantly improve the overall model [Δ*R*^2^ < 0.001, *F* (1,714) = 0.635, *p* = 0.426].

## Discussion

We examined the associations of coping using sex before UK lockdown (March 2020), and change in coping using sex scores during compared with pre-lockdown (retrospectively reported), with perceived or self-reported changes in health-related behaviors and mental health symptomatology in a UK community sample. In contrast to our hypotheses, greater coping using consensual sexual behaviors before lockdown was associated with a perceived overall improvement, rather than a worsening, in health-related behaviors. However, coping using sex before lockdown was not significantly associated with the number of health-related behaviors to worsen, and retrospectively reported change in coping using sex (two weeks during lockdown compared with the two-week period immediately preceding lockdown) did not predict changes in either health related behaviors or mental health symptomatology. In a series of exploratory analyses, higher levels of coping using sex before lockdown were associated with higher self-reported depression, anxiety, and stress scores during lockdown.

In addition, loneliness emerged as a significant predictor of negative mental health outcomes across the board, being related to both trait-levels of anxiety, depression, and stress during lockdown, and an increase in perceived mental health symptoms compared to before lockdown. Much of the research into the role of loneliness during the COVID-19 pandemic has focused on healthcare workers. For example, there are reports of increased loneliness during lockdown compared to before lockdown (39), and it has been shown that loneliness during lockdown was associated with poorer mental health (40). Our findings in this community survey study mirror these findings from samples of healthcare workers that highlight the importance of considering the experience of loneliness. In light of these findings, loneliness may represent a potential treatment target during times of limited social interaction, in order to prevent the onset of anxious and depressed feelings within the general population. A recent meta-analysis (41), which aimed to identify effective interventions to address feelings of loneliness that were feasible under the social distancing guidelines imposed during the COVID-19 pandemic, showed that mindfulness-based interventions (42, 43), weekly meditation classes (44), and laughter therapy (45) were all effective for improving loneliness.

Our findings suggest that participants who reported greater use of consensual sexual behaviors to cope before lockdown perceived that they had responded more adaptively in terms of, for example, improved sleep, eating more healthily, smoking less, and consuming less alcohol in the early weeks of lockdown. This pattern of findings may indicate that coping using sex was associated with greater reactivity to make changes to one's lifestyle in response to the UK lockdown, and in particular, with making changes toward a healthier lifestyle.

Although findings may appear to reflect improvements in health related behaviors, these apparent improvements may be indicative of changes in lifestyle that are known to be associated with poorer mental health. For example, depression has been associated with changes in sleep, resulting in both insomnia and hypersomnia (46, 47), and with both an increase and decrease in appetite (48). A change in self-perceived eating patterns toward more healthy eating can also be indicative of a restrictive diet, ritualized eating and rigid avoidance of “unhealthy” foods that is characteristic of some patterns of disordered eating (49). This interpretation is also supported by the finding that coping using sex prior to lockdown was associated with higher scores on the DASS-21, indicative of poorer mental health, including higher levels of depression, anxiety, and stress, during lockdown. The associations are in line with the known relationships of coping using sex with difficulties in emotion regulation (19).

It is possible that people who use consensual sexual behaviors to cope also engage in attempts to modify their lifestyle, including changing one's habits in terms of eating, sleeping, smoking, and drinking as a means of asserting control. This interpretation is consistent with reports that other coping strategies such as obsessive healthy eating may be employed in order to feel control in individuals with poor emotion regulation abilities (50). The use of sex as a means to cope with negative affective states has been associated with greater difficulties in emotion regulation (21), and with impairments in more typical emotion regulation strategies (e.g., cognitive reappraisal) that are associated with more positive and more adaptive outcomes (19, 40). The use of sex as a coping mechanism may therefore be most common in those who do not possess alternative, more adaptive strategies for emotion regulation. Emotion regulation strategies may therefore represent a potential treatment target, particularly during times of increased psychological strain such as the COVID-19 pandemic and consequent government-imposed lockdown. Strategies that aim to improve emotion regulation, including with a focus on reappraisal and acceptance-based approaches, may be affective in providing individuals with alternative, more adaptive, means of emotion regulation (51, 52). A recent study has identified that reappraisal interventions are effective in reducing negative emotions and increasing positive emotions specifically within the context of the COVID-19 pandemic (53).

Although our findings highlight the associations between coping using sex prior to lockdown and (mal)adaptive outcomes, they are subject to some limitations. First, the variance explained by the regression models examining perceived changes in health-related behaviors (R2 = 6.4%), and perceived changes in mental health symptomology (anxiety, R2 = 11.0%; depression, R2 = 18.3%), are relatively small. Our findings suggest that other factors beyond those included in the models should also be considered when examining changes in peoples' health-related behaviors and mental health symptomology over the course of the pandemic. For example, we did not collect information relating to past and current psychological and pharmacological treatment, which is likely to explain some of the variance in both mental health symptomology during lockdown and changes in mental health symptomology. However, the explained variance for the regression models predicting DASS-21 scores (e.g., total DASS score; R2 = 43.8%) were comparatively larger, which suggests that coping using sex and other variables included in the models might be more closely associated with absolute levels of mental health symptomology as compared to changes in mental health during compared to pre-lockdown.

Additionally, participants only reported measures at one time point during lockdown, and data corresponding to the 14-day period immediately preceding the introduction of lockdown were based on retrospective accounts. As such, data for this period are limited by the capacity to accurately report on previous states and it would be interesting to understand longer term changes that are associated with coping using sex.

There are also limitations associated with our scale asking about perceived changes in health-related behaviors and mental health, especially as participants reported perceived changes based on single items (e.g., anxiety) rather than across a range of items/symptoms (e.g., similar to the DASS-21), and these reports are unrevealing about overall frequency of behaviors or intensity of symptoms. As an illustrative example, a person may have perceived that their levels of physical activity increased due to engagement with a new weekly exercise class, but their overall level of activity may have decreased due to COVID-19 (e.g., reduction in active commuting or increase in sedentary time). Self-report biases may also vary based on participant characteristics. For example, a person experiencing high levels of stress may be more likely to perceive negative changes in lifestyle behaviors. Participants reported on their perceptions of how their behavior had changed. Measures that therefore rely on retrospective recall, are subjective (e.g., definitions of “healthy eating” will differ from person to person) and will be prone to bias.

Additionally, our sample was not overly representative of the wider UK population and was predominantly white. Education level was somewhat higher (65% educated to degree level and higher), most participants were female (67%), and the number of people with overweight and obesity were lower than in the United Kingdom, so results regarding ethnicity and education in particular should be interpreted cautiously. Likewise, those most affected by COVID-19 (i.e., experiencing stress and depression) may be less likely to have participated.

## Conclusions

Overall, our findings suggest that whilst coping using consensual sexual behaviors was not related with a perceived *worsening* in mental health symptomatology, greater coping using sex prior to lockdown was associated with higher levels of depression, anxiety and stress *during* lockdown. Greater coping using sex prior to lockdown was also associated with perceived changes in health-related behaviors, in the direction of increased healthy behaviors. Some studies have shown changes in sexual behaviors during lockdown. Our results suggest that where sexual behaviors are being used as a means to cope with negative or adverse situations, participants may be experiencing high levels of mental health symptomatology, including depression, anxiety, and stress. As such, it is important that future research continues to examine the long-term effects of coping using sex in the community both during and beyond the COVID-19 pandemic.

## Data Availability Statement

The datasets presented in this study can be found in online repositories. The names of the repository/repositories and accession number(s) can be found below: Open Science Framework–https://osf.io/vwshx/.

## Ethics Statement

The studies involving human participants were reviewed and approved by Liverpool University Ethics Committee. The patients/participants provided their written informed consent to participate in this study.

## Author Contributions

AJ: investigation. ND: formal analysis and writing—original draft. SG and ER: funding acquisition. SG, AJ, KU, CG, ER, and ND: conceptualization and writing—review and editing. All authors contributed to the article and approved the submitted version.

## Funding

Economic and Social Research Council, European Research Council, and University of Liverpool.

## Conflict of Interest

The authors declare that the research was conducted in the absence of any commercial or financial relationships that could be construed as a potential conflict of interest.

## Publisher's Note

All claims expressed in this article are solely those of the authors and do not necessarily represent those of their affiliated organizations, or those of the publisher, the editors and the reviewers. Any product that may be evaluated in this article, or claim that may be made by its manufacturer, is not guaranteed or endorsed by the publisher.
