# Supplementary material for: Coping Using Sex, Health-Related Behaviors, and Mental Health During COVID-19 Lockdown in the UK
Source: Front Psychiatry. 2022 May 24;13:880454. doi: 10.3389/fpsyt.2022.880454 (PMC9171108; doi:10.3389/fpsyt.2022.880454)
Supplement: Supplementary file 1 [file Data_Sheet_1.docx]

|  |  | **Loneliness** | **Social Distancing**  **(Physical)** | **CUS Before Lockdown**  **(Consent)** | **CUS During Lockdown**  **(Consent)** | **Perceived Change in HRB^a^** | **Number of HRB Worsened** | **Perceived Change in Anxiety** | **Perceived Change in Depression** | **Depression (DASS-21)** | **Anxiety (DASS-21)** | **Stress (DASS-21)** |
| --- | --- | --- | --- | --- | --- | --- | --- | --- | --- | --- | --- | --- |
| Loneliness |  | - |  |  |  |  |  |  |  |  |  |  |
| Social Distancing  (Physical) | Pearson’s r | -0.066 | - |  |  |  |  |  |  |  |  |  |
|  | p-value | 0.077 |  |  |  |  |  |  |  |  |  |  |
| CUSI Before Lockdown (Consent) | Pearson’s r | 0.078 | -0.095 | - |  |  |  |  |  |  |  |  |
|  | p-value | 0.035* | 0.011* |  |  |  |  |  |  |  |  |  |
| CUSI During Lockdown (Consent) | Pearson’s r | 0.089 | -0.085 | 0.880 | - |  |  |  |  |  |  |  |
|  | p-value | 0.017* | 0.022* | <.001*** |  |  |  |  |  |  |  |  |
| Perceived Change in HRB | Pearson’s r | -0.091 | 0.087 | 0.069 | 0.059 | - |  |  |  |  |  |  |
|  | p-value | 0.014* | 0.019* | 0.063 | 0.111 |  |  |  |  |  |  |  |
| Number of HRB Worsened | Pearson’s r | 0.181 | -0.076 | 0.013 | 0.035 | -0.730 | - |  |  |  |  |  |
|  | p-value | <.001*** | 0.040* | 0.720 | 0.348 | <.001*** |  |  |  |  |  |  |
| Perceived Change in Anxiety | Pearson’s r | 0.239 | 0.010 | 0.025 | 0.013 | -0.265 | 0.098 | - |  |  |  |  |
|  | p-value | <.001*** | 0.797 | 0.503 | 0.732 | <.001*** | 0.008** |  |  |  |  |  |
| Perceived Change in Depression | Pearson’s r | 0.375 | -0.010 | 0.040 | 0.017 | -0.091 | 0.191 | 0.561 | - |  |  |  |
|  | p-value | <.001*** | 0.794 | 0.284 | 0.651 | 0.014* | <.001*** | <.001*** |  |  |  |  |
| Depression (DASS-21) | Pearson’s r | 0.654 | -0.020 | 0.104 | 0.118 | -0.154 | 0.248 | 0.333 | 0.502 | - |  |  |
|  | p-value | <.001*** | 0.584 | 0.005** | 0.001** | <.001*** | <.001*** | <.001*** | <.001*** |  |  |  |
| Anxiety (DASS-21) | Pearson’s r | 0.434 | -0.076 | 0.094 | 0.078 | -0.078 | 0.190 | 0.250 | 0.406 | 0.643 | - |  |
|  | p-value | <.001*** | 0.041* | 0.011* | 0.036* | 0.035* | <.001*** | <.001*** | <.001*** | <.001*** |  |  |
| Stress (DASS-21) | Pearson’s r | 0.485 | -0.033 | 0.094 | 0.067 | -0.148 | 0.273 | 0.299 | 0.477 | 0.741 | 0.718 | - |
|  | p-value | <.001*** | 0.378 | 0.011* | 0.069 | <.001*** | <.001*** | <.001*** | <.001*** | <.001*** | <.001*** |  |

**Supplementary Materials 1** Table S1: Correlation Matrix

Note: CUSI = Coping Using Sex Inventory; HRB = Health Related Behaviors.

^a^Higher number indicates more positive change

*Indicates a significance level <.050

**Indicates a significance level < .010

***Indicates a significance level < .001
