# Supplementary material for: Coping Using Sex, Health-Related Behaviors, and Mental Health During COVID-19 Lockdown in the UK
Source: Front Psychiatry. 2022 May 24;13:880454. doi: 10.3389/fpsyt.2022.880454 (PMC9171108; doi:10.3389/fpsyt.2022.880454)
Supplement: Supplementary file 2 [file Data_Sheet_2.docx]

**Supplementary Materials 2**

**1 Perceived change in health-related behaviors**

The results for all models of the linear regression analysis for perceived change in health-related behaviors are shown in Table S1. Model one (*F*(8, 718) = 4.47, *p* < .001) explained 4.7% of the variance in perceived change in health-related behaviors. Model two explained 5.9% of the total variance, a statistically significant improvement (*ΔR^2^* = .011, *F*(2, 716) = 4.45, *p* = .015). Model three explained 6.4% of the total variance in perc3ived change in health-related behaviors, again a statistically significant improvement (*ΔR^2^* = .005, *F*(1, 715) = 4.17, *p* = .041). The inclusion of change in coping using sex scores in model four did not significantly improve the model (Δ*R^2^* < .001, *F*(1, 714) = 0.35, *p* = .558).

Table S1: Results of multiple linear regression on perceived change in health-related behaviors

| Predictor | Estimate | *SE* | *t* | *p* |
| --- | --- | --- | --- | --- |
| Model 1 |  |  |  |  |
| Gender: |  |  |  |  |
| male – female | -0.380 | 0.396 | -0.959 | 0.338 |
| Age | -0.041 | 0.019 | -2.087 | 0.037* |
| Ethnicity: |  |  |  |  |
| white – not white | -1.399 | 0.460 | -3.042 | 0.002** |
| Education |  |  |  |  |
| degree and above – lower than degree | -0.151 | 0.385 | -0.393 | 0.694 |
| Living Status: |  |  |  |  |
| alone – not alone | -1.165 | 0.605 | -1.926 | 0.055 |
| Previous Diagnosis of Psychiatric Condition: |  |  |  |  |
| yes – no | -1.174 | 0.400 | -2.937 | 0.003** |
| BMI | -0.043 | 0.032 | -1.351 | 0.177 |
| COVID-19 High Risk Health Group: |  |  |  |  |
| high risk condition – no high-risk condition | 0.239 | 0.481 | 0.496 | 0.620 |
| Model 2 |  |  |  |  |
| Gender: |  |  |  |  |
| male – female | -0.325 | 0.395 | -0.823 | 0.411 |
| Age | -0.048 | 0.020 | -2.416 | 0.016* |
| Ethnicity: |  |  |  |  |
| white – not white | -1.343 | 0.458 | -2.930 | 0.004** |
| Education: |  |  |  |  |
| degree and above – lower than degree | -0.176 | 0.383 | -0.460 | 0.646 |
| Living Status: |  |  |  |  |
| alone – not alone | -1.090 | 0.603 | -1.807 | 0.071 |
| Previous Diagnosis of Psychiatric Condition: |  |  |  |  |
| yes – no | -0.934 | 0.414 | -2.258 | 0.024** |
| BMI | -0.041 | 0.032 | -1.316 | 0.189 |
| COVID-19 High Risk Health Group: |  |  |  |  |
| high risk condition – no high-risk condition | 0.186 | 0.480 | 0.388 | 0.698 |
| Loneliness | -0.025 | 0.014 | -1.825 | 0.068 |
| Social Distancing | 0.695 | 0.324 | 2.143 | 0.032** |
| Model 3 |  |  |  |  |
| Gender: |  |  |  |  |
| male – female | -0.674 | 0.430 | -1.569 | 0.117 |
| Age | -0.037 | 0.020 | -1.805 | 0.071 |
| Ethnicity: |  |  |  |  |
| white – not white | -1.447 | 0.460 | -3.143 | 0.002** |
| Education: |  |  |  |  |
| degree and above – lower than degree | -0.200 | 0.382 | -0.523 | 0.601 |
| Living Status: |  |  |  |  |
| alone – not alone | -1.088 | 0.602 | -1.808 | 0.071 |
| Previous Diagnosis of Psychiatric Condition: |  |  |  |  |
| yes – no | -0.908 | 0.413 | -2.199 | 0.028** |
| BMI | -0.046 | 0.032 | -1.464 | 0.144 |
| COVID-19 High Risk Health Group: |  |  |  |  |
| high risk condition – no high-risk condition | 0.228 | 0.479 | 0.476 | 0.634 |
| Loneliness | -0.027 | 0.014 | -1.928 | 0.054 |
| Social Distancing | 0.745 | 0.325 | 2.296 | 0.022* |
| Coping Using Sex Prior to Lockdown | 0.088 | 0.043 | 2.042 | 0.041* |

*Indicates a significance level < .050

**Indicates a significance level < .010

***Indicates a significance level < .001

In an exploratory analysis, we replaced the addition of change in coping using sex in the final step, with the 2-way interaction of gender with coping using sex prior to lockdown. This step did not significantly improve the overall model (*ΔR^2^* < .001, *F*(1,714) = 0.011, *p* = .917).

**2 Number of health-related behaviors worsened**

The results for all models of the ordinal logistic regression analysis for the number of health-related behaviors for which participants reported a worsening are shown in Table S2. Model one (*χ^2^*(8) = 21.40, *p* = .005), explained 0.9% of the variance in the number of health-related behaviors worsened. Model two explained 1.6% of the total variance, a statistically significant improvement (*ΔR^2^_McF_* = 0.005, *χ^2^*(2) = 16.03, *p* < .001). The addition of coping using sex prior to lockdown (model 3; *χ^2^*(1) = 0.02, *p* = .884) and change in coping using sex (model 4; *χ^2^*(1) = 1.59, *p* = .208) did not significantly improve model fit.

Table S2: Results of ordinal logistic regression on no. health-related behaviors worsened

|  |  |  |  |  |  | 95% Confidence Interval | |
| --- | --- | --- | --- | --- | --- | --- | --- |
| Predictor | Estimate | *SE* | *Z* | *p* | Odds Ratio | Lower | Upper |
| Model 1 |  |  |  |  |  |  |  |
| Gender: |  |  |  |  |  |  |  |
| male – female | -0.037 | 0.147 | -0.248 | 0.804 | 0.964 | 0.722 | 1.287 |
| Age | -0.011 | 0.007 | -1.488 | 0.137 | 0.989 | 0.975 | 1.003 |
| Ethnicity: |  |  |  |  |  |  |  |
| white – not white | 0.322 | 0.168 | 1.914 | 0.056 | 1.380 | 0.993 | 1.920 |
| Education |  |  |  |  |  |  |  |
| degree and above – lower than degree | -0.162 | 0.144 | -1.123 | 0.261 | 0.851 | 0.641 | 1.128 |
| Living Status: |  |  |  |  |  |  |  |
| alone – not alone | 0.176 | 0.224 | 0.784 | 0.433 | 1.192 | 0.768 | 1.849 |
| Previous Diagnosis of Psychiatric Condition: |  |  |  |  |  |  |  |
| yes – no | 0.388 | 0.148 | 2.610 | 0.009 ** | 1.473 | 1.102 | 1.972 |
| BMI | 0.023 | 0.012 | 1.926 | 0.054 | 1.023 | 1.000 | 1.047 |
| COVID-19 High Risk Health Group: |  |  |  |  |  |  |  |
| high risk condition – no high-risk condition | -0.145 | 0.179 | -0.812 | 0.417 | 0.865 | 0.608 | 1.227 |
| Model 2 |  |  |  |  |  |  |  |
| Gender: |  |  |  |  |  |  |  |
| male – female | -0.032 | 0.148 | -0.218 | 0.827 | 0.968 | 0.724 | 1.294 |
| Age | -0.005 | 0.007 | -0.727 | 0.468 | 0.995 | 0.980 | 1.009 |
| Ethnicity: |  |  |  |  |  |  |  |
| white – not white | 0.286 | 0.169 | 1.691 | 0.091 | 1.331 | 0.956 | 1.855 |
| Education: |  |  |  |  |  |  |  |
| degree and above – lower than degree | -0.138 | 0.144 | -0.956 | 0.339 | 0.871 | 0.656 | 1.156 |
| Living Status: |  |  |  |  |  |  |  |
| alone – not alone | 0.172 | 0.224 | 0.769 | 0.442 | 1.188 | 0.765 | 1.842 |
| Previous Diagnosis of Psychiatric Condition: |  |  |  |  |  |  |  |
| yes – no | 0.219 | 0.155 | 1.414 | 0.157 | 1.245 | 0.919 | 1.689 |
| BMI | 0.020 | 0.012 | 1.703 | 0.088 | 1.020 | 0.997 | 1.044 |
| COVID-19 High Risk Health Group: |  |  |  |  |  |  |  |
| high risk condition – no high-risk condition | -0.106 | 0.179 | -0.590 | 0.555 | 0.900 | 0.632 | 1.278 |
| Loneliness | 0.019 | 0.005 | 3.616 | <.001 *** | 1.019 | 1.009 | 1.030 |
| Social Distancing | -0.167 | 0.127 | -1.316 | 0.188 | 0.846 | 0.660 | 1.085 |

*Indicates a significance level < .050

**Indicates a significance level < .010

***Indicates a significance level < .001

In an exploratory analysis, we replaced the addition of change in coping using sex in the final step, with the 2-way interaction of gender with coping using sex prior to lockdown. This step did not significantly improve the overall model (*ΔR^2^_McF_* = .016, *χ^2^*(1) = 0.047, *p* = .828).

**3 Confirmatory factor analysis for health-related behaviors**

Confirmatory factor analysis (CFA) was used to investigate the internal consistency of the health-related behaviour questionnaire. Items with low item-total correlations and low item loadings were removed from the questionnaire prior to performing further regression analyses. All regression analyses were then repeated, using only the health-related behaviors that remained following confirmatory factor analysis.

For the CFA, the results of the Chi-Square Test of Goodness of Fit were consistent with the six health related behaviour items measuring a single underlying construct (*χ^2^*(9,765) = 194, *p* < .001). The Comparative Fit Index (CFI) and Root Mean Square Error of Approximation (RMSEA) values (*CFI* = 0.61, *RMSEA* = 0.16), however, were not consistent with a single factor model. The factor loadings indicated that the item *smoked* (β = 0.21, *p* = .001) loaded least well onto the single factor (for all other items, *p* < .001). Table S3 shows the results of the CFA with this item removed. The Chi-Square Test of Goodness of Fit was not consistent with a single factor (*χ^2^*(5,765) = 38.3, *p* < .001), however the CFI value (*CFI* = 0.90) was consistent with a single factor and the RMSEA value was approaching the recommended cut-off for acceptable fit (*RMSEA* = .093). Given, the small number of items, we considered these results as indicative that our measure was indeed measuring a single underlying construct.

Table S3: Results of Confirmatory Factor Analysis for health-related behaviors

|  |  |  |  |  |  |  |  |  |  |  |  |  |
| --- | --- | --- | --- | --- | --- | --- | --- | --- | --- | --- | --- | --- |
| Factor | | Indicator | | Estimate | | *SE* | | *Z* | | *p* | | |
| Factor 1 |  | Exercised |  | 0.797 |  | 0.084 |  | 9.47 |  | < .001 |  |  |
|  |  | Slept |  | 0.457 |  | 0.066 |  | 6.89 |  | < .001 |  |  |
|  |  | Eaten Healthily |  | 1.298 |  | 0.099 |  | 13.13 |  | < .001 |  |  |
|  |  | Binged Food |  | 0.455 |  | 0.056 |  | 8.16 |  | < .001 |  |  |
|  |  | Drank Alcohol |  | 0.212 |  | 0.064 |  | 3.31 |  | < .001 |  |  |

**4 Perceived change in health-related behaviours (smoking item removed)**

The results of the linear regression analysis for perceived change in health-related behaviors (smoking item removed) are shown in Table S4. Model one (*F*(8,718) = 3.44, *p* < .001) explained 3.7% of the variance in perceived change in health-related behaviors. Model two explained 5.2% of the total variance, a statistically significant improvement (*ΔR^2^* = .015, *F*(2,716) = 5.50, *p* = .004). Model three explained 6.0% of the total variance in total health-related behaviour score, again a statistically significant improvement (*ΔR^2^* = .008, *F*(1,715) = 6.45, *p* = .011). Adding the change in coping using sex in model four did not significantly improve the model (*ΔR^2^* < .001, *F*(1,714) = 0.16, *p* = .694).

Based on the most parsimonious model (model 3), adhering to social distancing and greater levels of coping using sex prior to lockdown predicted more positive change in health-related behaviors, whilst being white and being lonelier predicted a perceived worsening in health-related behaviors.

Table S4: Results of multiple linear regression on perceived change in health-related behaviors (minus smoking)

| Predictor | Estimate | *SE* | *t* | *p* |
| --- | --- | --- | --- | --- |
| Model 1 |  |  |  |  |
| Gender: |  |  |  |  |
| male – female | -0.071 | 0.355 | -0.201 | 0.841 |
| Age | -0.030 | 0.017 | -1.716 | 0.087 |
| Ethnicity: |  |  |  |  |
| white – not white | -0.952 | 0.412 | -2.310 | 0.021* |
| Education |  |  |  |  |
| degree and above – lower than degree | 0.062 | 0.344 | 0.180 | 0.857 |
| Living Status: |  |  |  |  |
| alone – not alone | -0.957 | 0.542 | -1.767 | 0.078 |
| Previous Diagnosis of Psychiatric Condition: |  |  |  |  |
| yes – no | -0.901 | 0.358 | -2.517 | 0.012* |
| BMI | -0.045 | 0.028 | -1.579 | 0.115 |
| COVID-19 High Risk Health Group: |  |  |  |  |
| high risk condition – no high-risk condition | 0.175 | 0.431 | 0.405 | 0.685 |
| Model 2 |  |  |  |  |
| Gender: |  |  |  |  |
| male – female | -0.028 | 0.353 | -0.078 | 0.938 |
| Age | -0.039 | 0.018 | -2.205 | 0.028* |
| Ethnicity: |  |  |  |  |
| white – not white | -0.885 | 0.410 | -2.159 | 0.031* |
| Education: |  |  |  |  |
| degree and above – lower than degree | 0.030 | 0.343 | 0.088 | 0.930 |
| Living Status: |  |  |  |  |
| alone – not alone | -0.889 | 0.539 | -1.648 | 0.100 |
| Previous Diagnosis of Psychiatric Condition: |  |  |  |  |
| yes – no | -0.610 | 0.370 | -1.650 | 0.099 |
| BMI | -0.042 | 0.028 | -1.492 | 0.136 |
| COVID-19 High Risk Health Group: |  |  |  |  |
| high risk condition – no high-risk condition | 0.114 | 0.429 | 0.265 | 0.791 |
| Loneliness | -0.033 | 0.012 | -2.624 | 0.009** |
| Social Distancing | 0.542 | 0.290 | 1.869 | 0.062 |
| Model 3 |  |  |  |  |
| Gender: |  |  |  |  |
| male – female | -0.415 | 0.383 | -1.082 | 0.279 |
| Age | -0.027 | 0.018 | -1.474 | 0.141 |
| Ethnicity: |  |  |  |  |
| white – not white | -1.000 | 0.411 | -2.433 | 0.015* |
| Education: |  |  |  |  |
| degree and above – lower than degree | 0.004 | 0.341 | 0.010 | 0.992 |
| Living Status: |  |  |  |  |
| alone – not alone | -0.886 | 0.537 | -1.650 | 0.099 |
| Previous Diagnosis of Psychiatric Condition: |  |  |  |  |
| yes – no | -0.582 | 0.369 | -1.579 | 0.115 |
| BMI | -0.047 | 0.028 | -1.679 | 0.094 |
| COVID-19 High Risk Health Group: |  |  |  |  |
| high risk condition – no high-risk condition | 0.161 | 0.428 | 0.375 | 0.707 |
| Loneliness | -0.034 | 0.012 | -2.757 | 0.006** |
| Social Distancing | 0.598 | 0.290 | 2.062 | 0.040* |
| Coping Using Sex Prior to Lockdown | 0.098 | 0.039 | 2.540 | 0.011* |

*Indicates a significance level < .050

**Indicates a significance level < .010

***Indicates a significance level < .001

In an exploratory analysis, we replaced the addition of change in coping using sex in the final step, with the 2-way interaction of gender with coping using sex prior to lockdown. This step did not significantly improve the overall model (*ΔR^2^* < .001, *F*(1,714) = 0.009, *p* = .923).

**5 Number of health-related behaviors worsened (smoking item removed)**

The results of an ordinal logistic regression for the number of health-related behaviors for which participants reported a decline are shown in Table S5. Model one (*χ^2^*(8) = 17.57, *p* = .025) explained 0.8% of the variance in the number of health-related behaviors worsened. Model two explained 1.4% of the total variance, a statistically significant improvement (*ΔR^2^_McF_* = 0.006, *χ^2^*(2) = 15.19, *p* < .001). The addition of coping using sex prior to lockdown (model 3; *χ^2^*(1) = 0.50, *p* = .481) and change in coping using sex (model 4; *χ^2^*(1) = 1.24, *p* = .266) did not significantly improve model fit.

Based on the most parsimonious model (model two), only loneliness was a statistically significant predictor of the number of health-related behaviors to decline; greater loneliness predicted a decline in a greater number of health-related behaviors.

Table S5: Results of ordinal logistic regression on no. health-related behaviors worsened (minus smoking)

|  |  |  |  |  |  | 95% Confidence Interval | |
| --- | --- | --- | --- | --- | --- | --- | --- |
| Predictor | Estimate | *SE* | *Z* | *p* | Odds Ratio | Lower | Upper |
| Model 1 |  |  |  |  |  |  |  |
| Gender: |  |  |  |  |  |  |  |
| male – female | -0.080 | 0.148 | -0.540 | 0.589 | 0.923 | 0.691 | 1.234 |
| Age | -0.011 | 0.007 | -1.510 | 0.131 | 0.989 | 0.975 | 1.003 |
| Ethnicity: |  |  |  |  |  |  |  |
| white – not white | 0.302 | 0.169 | 1.790 | 0.073 | 1.352 | 0.972 | 1.883 |
| Education |  |  |  |  |  |  |  |
| degree and above – lower than degree | -0.090 | 0.144 | -0.625 | 0.532 | 0.914 | 0.689 | 1.212 |
| Living Status: |  |  |  |  |  |  |  |
| alone – not alone | 0.216 | 0.224 | 0.964 | 0.335 | 1.241 | 0.799 | 1.924 |
| Previous Diagnosis of Psychiatric Condition: |  |  |  |  |  |  |  |
| yes – no | 0.313 | 0.149 | 2.102 | 0.036* | 1.367 | 1.021 | 1.831 |
| BMI | 0.023 | 0.012 | 1.999 | 0.046* | 1.024 | 1.000 | 1.048 |
| COVID-19 High Risk Health Group: |  |  |  |  |  |  |  |
| high risk condition – no high-risk condition | -0.179 | 0.180 | -0.990 | 0.322 | 0.836 | 0.587 | 1.190 |
| Model 2 |  |  |  |  |  |  |  |
| Gender: |  |  |  |  |  |  |  |
| male – female | -0.071 | 0.149 | -0.479 | 0.632 | 0.931 | 0.695 | 1.247 |
| Age | -0.005 | 0.007 | -0.737 | 0.461 | 0.995 | 0.980 | 1.009 |
| Ethnicity: |  |  |  |  |  |  |  |
| white – not white | 0.271 | 0.169 | 1.600 | 0.109 | 1.311 | 0.941 | 1.829 |
| Education: |  |  |  |  |  |  |  |
| degree and above – lower than degree | -0.066 | 0.145 | -0.459 | 0.646 | 0.936 | 0.705 | 1.243 |
| Living Status: |  |  |  |  |  |  |  |
| alone – not alone | 0.219 | 0.224 | 0.979 | 0.328 | 1.245 | 0.801 | 1.932 |
| Previous Diagnosis of Psychiatric Condition: |  |  |  |  |  |  |  |
| yes – no | 0.142 | 0.156 | 0.914 | 0.361 | 1.153 | 0.849 | 1.565 |
| BMI | 0.021 | 0.012 | 1.754 | 0.079 | 1.021 | 0.998 | 1.045 |
| COVID-19 High Risk Health Group: |  |  |  |  |  |  |  |
| high risk condition – no high-risk condition | -0.139 | 0.181 | -0.767 | 0.443 | 0.871 | 0.610 | 1.240 |
| Loneliness | 0.020 | 0.005 | 3.806 | < .001*** | 1.020 | 1.010 | 1.031 |
| Social Distancing | -0.056 | 0.124 | -0.452 | 0.651 | 0.945 | 0.740 | 1.206 |

*Indicates a significance level < .050

**Indicates a significance level < .010

***Indicates a significance level < .001

In an exploratory analysis, we replaced the addition of change in coping using sex in the final step, with the 2-way interaction of gender with coping using sex prior to lockdown. This step did not significantly improve the overall model (*ΔR^2^_McF_* = .014, *χ^2^*(1) = 0.058, *p* = .810).
