# Supplementary material for: Coping Using Sex, Health-Related Behaviors, and Mental Health During COVID-19 Lockdown in the UK
Source: Front Psychiatry. 2022 May 24;13:880454. doi: 10.3389/fpsyt.2022.880454 (PMC9171108; doi:10.3389/fpsyt.2022.880454)
Supplement: Supplementary file 3 [file Data_Sheet_3.docx]

**Supplementary Materials 3**

**1 Perceived change in mental health**

Examination of the descriptive statistics for perceived change in mental health for each of the four mental health items suggested that an unexpectedly large proportion of our sample responded ‘*a lot less than unusual*’ to the suicidal thoughts and self-harmed items. These results imply that prior to the COVID-19 lockdown, 27.8% of our sample (*N* = 213) experienced suicidal thoughts, and 31.2% of our sample (*N* = 239) engaged in self harm. These figures represent a tenfold and a fivefold increase, respectively, on the expected proportion of participants who experienced suicidal thoughts (43) or engaged in self harm (44) in general population surveys in the UK. Because of the problematic nature of these responses, we diverged from our preregistered analyses to focus on scores for the remaining anxiety and the depression items individually using linear regression.

- 1. **Anxiety Item**

The results for all models of the multiple linear regression for anxiety are shown in Table S1. Model one (*F*(8, 718) = 6.76, *p* <.001) explained 7.0% of the variance in perceived change in anxiety. Model two explained 11.0% of the total variance, a statistically significant improvement (*ΔR^2^* = .040, *F*(2,716) = 16.25, *p* < .001). The addition of coping using sex prior to lockdown (model 3; *ΔR^2^* = .002, *F*(1,715) = 1.81, *p* = .179) and change in coping using sex (model 4; *ΔR^2^* = .002, *F*(1,714) = 1.61, *p* = .204) did not significantly improve model fit.

Table S1: Results of multiple linear regression on change in anxiety

| Predictor | Estimate | *SE* | *t* | *p* |
| --- | --- | --- | --- | --- |
| Model 1 |  |  |  |  |
| Gender: |  |  |  |  |
| male – female | -0.519 | 0.105 | -4.927 | < .001*** |
| Age | -0.019 | 0.005 | -3.585 | < .001*** |
| Ethnicity: |  |  |  |  |
| white – not white | 0.176 | 0.122 | 1.440 | 0.150 |
| Education |  |  |  |  |
| degree and above – lower than degree | 0.088 | 0.102 | 0.863 | 0.388 |
| Living Status: |  |  |  |  |
| alone – not alone | -0.097 | 0.161 | -0.601 | 0.548 |
| Previous Diagnosis of Psychiatric Condition: |  |  |  |  |
| yes – no | 0.246 | 0.106 | 2.316 | 0.021* |
| BMI | 0.004 | 0.008 | 0.487 | 0.627 |
| COVID-19 High Risk Health Group: |  |  |  |  |
| high risk condition – no high-risk condition | 0.047 | 0.128 | 0.371 | 0.711 |
| Model 2 |  |  |  |  |
| Gender: |  |  |  |  |
| male – female | -0.525 | 0.103 | -5.084 | < .001*** |
| Age | -0.013 | 0.005 | -2.527 | 0.012* |
| Ethnicity: |  |  |  |  |
| white – not white | 0.138 | 0.120 | 1.154 | 0.249 |
| Education: |  |  |  |  |
| degree and above – lower than degree | 0.108 | 0.100 | 1.079 | 0.281 |
| Living Status: |  |  |  |  |
| alone – not alone | -0.118 | 0.158 | -0.747 | 0.455 |
| Previous Diagnosis of Psychiatric Condition: |  |  |  |  |
| yes – no | 0.079 | 0.108 | 0.729 | 0.466 |
| BMI | 0.002 | 0.008 | 0.199 | 0.842 |
| COVID-19 High Risk Health Group: |  |  |  |  |
| high risk condition – no high-risk condition | 0.080 | 0.126 | 0.640 | 0.522 |
| Loneliness | 0.020 | 0.004 | 5.588 | < .001*** |
| Social Distancing | -0.067 | 0.085 | -0.792 | 0.429 |

*Indicates a significance level < .050

**Indicates a significance level < .010

***Indicates a significance level < .001

In an exploratory analysis, we replaced the addition of change in coping using sex in the final step, with the 2-way interaction of gender with coping using sex prior to lockdown. This step did not significantly improve the overall model (*ΔR^2^* < .001, *F*(1,714) = 0.043, *p* = .836).

- 1. **Depression Item**

The results for all models of the multiple linear regression for depression are shown in Table S2. Model one (*F*(8,718) = 7.48, *p* <.001) explained 7.7% of the variance in perceived change in depression. Model two explained 18.3% of the total variance, a statistically significant improvement (*ΔR^2^* = .106, *F*(2,716) = 46.66, *p* < .001). The addition of coping using sex prior to lockdown (model 3; *ΔR^2^* = .002, *F*(1,715) = 1.39, *p* = .238) and change in coping using sex (model 4; *ΔR^2^* < .001, *F*(1,714) = 0.14, *p* = .710) did not significantly improve model fit.

Table S2: Results of multiple linear regression on change in depression

| Predictor | Estimate | *SE* | *t* | *p* |
| --- | --- | --- | --- | --- |
| Model 1 |  |  |  |  |
| Gender: |  |  |  |  |
| male – female | -0.294 | 0.103 | -2.841 | 0.005** |
| Age | -0.022 | 0.005 | -4.398 | < .001*** |
| Ethnicity: |  |  |  |  |
| white – not white | 0.375 | 0.120 | 3.122 | 0.002** |
| Education |  |  |  |  |
| degree and above – lower than degree | <.001 | 0.100 | <.001 | 0.999 |
| Living Status: |  |  |  |  |
| alone – not alone | 0.132 | 0.158 | 0.838 | 0.402 |
| Previous Diagnosis of Psychiatric Condition: |  |  |  |  |
| yes – no | 0.403 | 0.104 | 3.861 | < .001*** |
| BMI | 0.009 | 0.008 | 1.122 | 0.262 |
| COVID-19 High Risk Health Group: |  |  |  |  |
| high risk condition – no high-risk condition | -0.076 | 0.126 | -0.604 | 0.546 |
| Model 2 |  |  |  |  |
| Gender: |  |  |  |  |
| male – female | -0.293 | 0.098 | -2.999 | 0.003** |
| Age | -0.013 | 0.005 | -2.749 | 0.006** |
| Ethnicity: |  |  |  |  |
| white – not white | 0.315 | 0.113 | 2.786 | 0.005** |
| Education: |  |  |  |  |
| degree and above – lower than degree | 0.032 | 0.095 | 0.344 | 0.731 |
| Living Status: |  |  |  |  |
| alone – not alone | 0.110 | 0.149 | 0.741 | 0.459 |
| Previous Diagnosis of Psychiatric Condition: |  |  |  |  |
| yes – no | 0.138 | 0.102 | 1.349 | 0.178 |
| BMI | 0.005 | 0.008 | 0.616 | 0.538 |
| COVID-19 High Risk Health Group: |  |  |  |  |
| high risk condition – no high-risk condition | -0.025 | 0.118 | -0.213 | 0.832 |
| Loneliness | 0.033 | 0.003 | 9.660 | < .001*** |
| Social Distancing | 0.046 | 0.080 | 0.570 | 0.569 |

*Indicates a significance level < .050

**Indicates a significance level < .010

***Indicates a significance level < .001

In an exploratory analysis, we replaced the addition of change in coping using sex in the final step, with the 2-way interaction of gender with coping using sex prior to lockdown. This step did not significantly improve the overall model (*ΔR^2^* < .001, *F*(1,714) = 0.635, *p* = .426).

1. **DASS-21 Total Score**

The results for all models of the linear regression analysis for depression during lockdown are shown in Table S3. Model one (*F*(8,717) = 19.53, *p* < .001) explained 17.9% of the variance in total DASS score during lockdown. Model two explained 41.8% of the total variance, a statistically significant improvement (*ΔR^2^* = .240, *F*(2,715) = 51.44, *p* < .001). Model three explained 42.8% of the total variance in total DASS score during lockdown, again a statistically significant improvement (*ΔR^2^* = .009, *F*(1,714) = 48.51, *p* = .002). Adding the change in coping using sex in model four did not significantly improve the overall model (*ΔR^2^* < .001, *F*(1,713) = 1.44, *p* = .624).

Based on the most parsimonious model (model 3), being male predicted lower total DASS score, whilst having a psychiatric condition, being more lonely and greater use of sex to cope prior to lockdown predicted greater total DASS score.

Table S3: Results of multiple linear regression on DASS-21 total score during lockdown

| Predictor | Estimate | *SE* | *t* | *p* |
| --- | --- | --- | --- | --- |
| Model 1 |  |  |  |  |
| Gender: |  |  |  |  |
| male – female | -2.046 | 0.898 | -2.278 | 0.023* |
| Age | -0.264 | 0.044 | -5.982 | < .001*** |
| Ethnicity: |  |  |  |  |
| white – not white | 1.576 | 1.044 | 1.509 | 0.132 |
| Education |  |  |  |  |
| degree and above – lower than degree | 0.228 | 0.872 | 0.262 | 0.794 |
| Living Status: |  |  |  |  |
| alone – not alone | -1.130 | 1.371 | -0.824 | 0.410 |
| Previous Diagnosis of Psychiatric Condition: |  |  |  |  |
| yes – no | 8.714 | 0.906 | 9.619 | < .001*** |
| BMI | 0.108 | 0.072 | 1.511 | 0.131 |
| COVID-19 High Risk Health Group: |  |  |  |  |
| high risk condition – no high-risk condition | -1.352 | 1.091 | -1.240 | 0.215 |
| Model 2 |  |  |  |  |
| Gender: |  |  |  |  |
| male – female | -2.067 | 0.758 | -2.725 | 0.007** |
| Age | -0.140 | 0.038 | -3.691 | <.001*** |
| Ethnicity: |  |  |  |  |
| white – not white | 0.702 | 0.881 | 0.797 | 0426 |
| Education: |  |  |  |  |
| degree and above – lower than degree | 0.691 | 0.735 | 0.940 | 0.347 |
| Living Status: |  |  |  |  |
| alone – not alone | -1.515 | 1.157 | -1.309 | 0.191 |
| Previous Diagnosis of Psychiatric Condition: |  |  |  |  |
| yes – no | 5.019 | 0.793 | 6.326 | < .001*** |
| BMI | 0.047 | 0.060 | 0.784 | 0.433 |
| COVID-19 High Risk Health Group: |  |  |  |  |
| high risk condition – no high-risk condition | -0.639 | 0.920 | -0.695 | 0.487 |
| Loneliness | 0.456 | 0.027 | 17.106 | < .001*** |
| Social Distancing | -0.207 | 0.622 | -0.333 | 0.739 |
| Model 3 |  |  |  |  |
| Gender: |  |  |  |  |
| male – female | -3.185 | 0.821 | -3.877 | <.001*** |
| Age | -0.105 | 0.039 | -2.708 | 0.007 |
| Ethnicity: |  |  |  |  |
| white – not white | 0.384 | 0.880 | 0.437 | 0.663 |
| Education: |  |  |  |  |
| degree and above – lower than degree | 0.611 | 0.730 | 0.837 | 0.403 |
| Living Status: |  |  |  |  |
| alone – not alone | -1.505 | 1.148 | -1.310 | 0.191 |
| Previous Diagnosis of Psychiatric Condition: |  |  |  |  |
| yes – no | 5.103 | 0.788 | 6.476 | < .001*** |
| BMI | 0.033 | 0.060 | 0.547 | 0.585 |
| COVID-19 High Risk Health Group: |  |  |  |  |
| high risk condition – no high-risk condition | -0.505 | 0.914 | -0.552 | 0.581 |
| Loneliness | 0.452 | 0.027 | 17.030 | < .001*** |
| Social Distancing | -0.049 | 0.619 | -0.079 | 0.937 |
| Coping Using Sex Prior to Lockdown | 0.281 | 0.082 | 3.403 | 0.001*** |

*Indicates a significance level < .050

**Indicates a significance level < .010

***Indicates a significance level < .001

In an exploratory analysis, we replaced the addition of change in coping using sex in the final step, with the 2-way interaction of gender with coping using sex prior to lockdown. This step did not significantly improve the overall model (*ΔR^2^* < .002, *F*(1,713) = 2.383, *p* = .123).

- 1. **Depression subscale of the DASS-21**

The results for all models of the linear regression analysis for depression during lockdown are shown in Table S4. Model one (*F*(8,718) = 16.24, *p* < .001) explained 15.3% of the variance in depression during lockdown. Model two explained 46.4% of the total variance, a statistically significant improvement (*ΔR^2^* = .311, *F*(2,716) = 207.73, *p* < .001). Model three explained 47.1% of the total variance in depression during lockdown, again a statistically significant improvement (*ΔR^2^* = .007, *F*(1,715) = 9.44, *p* = .002). Adding the change in coping using sex in model four did not significantly improve the overall model (*ΔR^2^* < .001, *F*(1,714) = 1.44, *p* = .230).

Based on the most parsimonious model (model 3), being male predicted lower levels of depression, whilst having a psychiatric condition, being more lonely and greater use of sex to cope prior to lockdown predicted greater levels of depression during lockdown.

Table S4: Results of multiple linear regression on DASS-21 depression during lockdown

| Predictor | Estimate | *SE* | *t* | *p* |
| --- | --- | --- | --- | --- |
| Model 1 |  |  |  |  |
| Gender: |  |  |  |  |
| male – female | -0.639 | 0.396 | -1.615 | 0.107 |
| Age | -0.106 | 0.019 | -5.428 | < .001*** |
| Ethnicity: |  |  |  |  |
| white – not white | 0.519 | 0.460 | 1.128 | 0.260 |
| Education |  |  |  |  |
| degree and above – lower than degree | 0.132 | 0.384 | 0.343 | 0.732 |
| Living Status: |  |  |  |  |
| alone – not alone | 0.384 | 0.605 | 0.635 | 0.526 |
| Previous Diagnosis of Psychiatric Condition: |  |  |  |  |
| yes – no | 3.619 | 0.400 | 9.055 | < .001*** |
| BMI | 0.038 | 0.032 | 1.219 | 0.223 |
| COVID-19 High Risk Health Group: |  |  |  |  |
| high risk condition – no high-risk condition | -0.719 | 0.481 | -1.494 | 0.136 |
| Model 2 |  |  |  |  |
| Gender: |  |  |  |  |
| male – female | -0.640 | 0.316 | -2.027 | 0.043* |
| Age | -0.044 | 0.016 | -2.817 | 0.005** |
| Ethnicity: |  |  |  |  |
| white – not white | 0.112 | 0.367 | 0.304 | 0.761 |
| Education: |  |  |  |  |
| degree and above – lower than degree | 0.353 | 0.306 | 1.153 | 0.249 |
| Living Status: |  |  |  |  |
| alone – not alone | 0.225 | 0.482 | 0.467 | 0.641 |
| Previous Diagnosis of Psychiatric Condition: |  |  |  |  |
| yes – no | 1.802 | 0.331 | 5.447 | < .001*** |
| BMI | 0.008 | 0.025 | 0.331 | 0.741 |
| COVID-19 High Risk Health Group: |  |  |  |  |
| high risk condition – no high-risk condition | -0.370 | 0.384 | -0.965 | 0.335 |
| Loneliness | 0.226 | 0.011 | 20.378 | < .001*** |
| Social Distancing | 0.200 | 0.259 | 0.769 | 0.442 |
| Model 3 |  |  |  |  |
| Gender: |  |  |  |  |
| male – female | -1.059 | 0.342 | -3.093 | 0.002** |
| Age | -0.031 | 0.016 | -1.934 | 0.053 |
| Ethnicity: |  |  |  |  |
| white – not white | -0.012 | 0.367 | -0.033 | 0.974 |
| Education: |  |  |  |  |
| degree and above – lower than degree | 0.325 | 0.305 | 1.066 | 0.287 |
| Living Status: |  |  |  |  |
| alone – not alone | 0.227 | 0.479 | 0.474 | 0.635 |
| Previous Diagnosis of Psychiatric Condition: |  |  |  |  |
| yes – no | 1.833 | 0.329 | 5.570 | < .001*** |
| BMI | 0.003 | 0.025 | 0.108 | 0.914 |
| COVID-19 High Risk Health Group: |  |  |  |  |
| high risk condition – no high-risk condition | -0.320 | 0.382 | -0.838 | 0.403 |
| Loneliness | 0.225 | 0.011 | 20.320 | < .001*** |
| Social Distancing | 0.260 | 0.259 | 1.004 | 0.316 |
| Coping Using Sex Prior to Lockdown | 0.106 | 0.034 | 3.072 | 0.002** |

*Indicates a significance level < .050

**Indicates a significance level < .010

***Indicates a significance level < .001

In an exploratory analysis, we replaced the addition of change in coping using sex in the final step, with the 2-way interaction of gender with coping using sex prior to lockdown. This step did not significantly improve the overall model (*ΔR^2^* < .001, *F*(1,714) = 0.789, *p* = .375).

- 1. **Anxiety subscale of the DASS-21**

The results for all models of the linear regression analysis for anxiety during lockdown are shown in Table S5. Model one (*F*(8,718) = 17.14, *p* < .001) explained 16.0% of the variance in anxiety during lockdown. Model two explained 26.7% of the total variance, a statistically significant improvement (*ΔR^2^* = .106, *F*(2,716) = 51.36, *p* < .001). Model three explained 27.2% of the total variance in anxiety during lockdown, again a statistically significant improvement (*ΔR^2^* = .006, *F*(1,715) = 5.69, *p* = .017). Adding the change in coping using sex in model four did not significantly improve the overall model (*ΔR^2^* = .001, *F*(1,714) = 1.38, *p* = .241).

Based on the most parsimonious model (model 3), being male and being younger predicted lower levels of anxiety, whilst having a psychiatric condition, being more lonely and greater use of sex to cope prior to lockdown predicted greater levels of anxiety during lockdown.

Table S5: Results of multiple linear regression on DASS-21 anxiety during lockdown

| Predictor | Estimate | *SE* | *t* | *p* |
| --- | --- | --- | --- | --- |
| Model 1 |  |  |  |  |
| Gender: |  |  |  |  |
| male – female | -0.384 | 0.259 | -1.484 | 0.138 |
| Age | -0.077 | 0.013 | -6.026 | < .001*** |
| Ethnicity: |  |  |  |  |
| white – not white | -0.011 | 0.300 | -0.038 | 0.970 |
| Education |  |  |  |  |
| degree and above – lower than degree | 0.024 | 0.251 | 0.097 | 0.923 |
| Living Status: |  |  |  |  |
| alone – not alone | -0.256 | 0.395 | -0.648 | 0.517 |
| Previous Diagnosis of Psychiatric Condition: |  |  |  |  |
| yes – no | 2.338 | 0.261 | 8.958 | < .001*** |
| BMI | 0.040 | 0.021 | 1.937 | 0.053 |
| COVID-19 High Risk Health Group: |  |  |  |  |
| high risk condition – no high-risk condition | -0.046 | 0.314 | -0.148 | 0.882 |
| Model 2 |  |  |  |  |
| Gender: |  |  |  |  |
| male – female | -0.415 | 0.242 | -1.710 | 0.088 |
| Age | -0.054 | 0.012 | -4.422 | < .001*** |
| Ethnicity: |  |  |  |  |
| white – not white | -0.169 | 0.281 | -0.602 | 0.548 |
| Education: |  |  |  |  |
| degree and above – lower than degree | 0.107 | 0.235 | 0.456 | 0.648 |
| Living Status: |  |  |  |  |
| alone – not alone | -0.348 | 0.370 | -0.940 | 0.348 |
| Previous Diagnosis of Psychiatric Condition: |  |  |  |  |
| yes – no | 1.638 | 0.254 | 6.452 | < .001*** |
| BMI | 0.030 | 0.019 | 1.544 | 0.123 |
| COVID-19 High Risk Health Group: |  |  |  |  |
| high risk condition – no high-risk condition | 0.092 | 0.294 | 0.311 | 0.756 |
| Loneliness | 0.085 | 0.009 | 9.936 | < .001*** |
| Social Distancing | -0.326 | 0.199 | -1.635 | 0.102 |
| Model 3 |  |  |  |  |
| Gender: |  |  |  |  |
| male – female | -0.665 | 0.263 | -2.523 | 0.012* |
| Age | -0.046 | 0.013 | -3.661 | < .001*** |
| Ethnicity: |  |  |  |  |
| white – not white | -0.243 | 0.282 | -0.862 | 0.389 |
| Education: |  |  |  |  |
| degree and above – lower than degree | 0.090 | 0.234 | 0.385 | 0.701 |
| Living Status: |  |  |  |  |
| alone – not alone | -0.346 | 0.369 | -0.939 | 0.348 |
| Previous Diagnosis of Psychiatric Condition: |  |  |  |  |
| yes – no | 1.656 | 0.253 | 6.543 | < .001*** |
| BMI | 0.026 | 0.019 | 1.371 | 0.171 |
| COVID-19 High Risk Health Group: |  |  |  |  |
| high risk condition – no high-risk condition | 0.122 | 0.294 | 0.414 | 0.679 |
| Loneliness | 0.084 | 0.009 | 9.838 | < .001*** |
| Social Distancing | -0.290 | 0.199 | -1.455 | 0.146 |
| Coping Using Sex Prior to Lockdown | 0.063 | 0.026 | 2.386 | 0.017* |

*Indicates a significance level < .050

**Indicates a significance level < .010

***Indicates a significance level < .001

In an exploratory analysis, we replaced the addition of change in coping using sex in the final step, with the 2-way interaction of gender with coping using sex prior to lockdown. This step did not significantly improve the overall model (*ΔR^2^* < .001, *F*(1,714) = 0.317, *p* = .574).

- 1. **Stress subscale of the DASS-21**

The results for all models of the linear regression analysis for stress during lockdown are shown in Table S6. Model one (*F*(8,717) = 14.30, *p* < .001) explained 37.1% of the variance in stress during lockdown. Model two explained 54.0% of the total variance, a statistically significant improvement (*ΔR^2^* = .154, *F*(2,715) = 77.88, *p* < .001). Model three explained 54.9% of the total variance in stress during lockdown, again a statistically significant improvement (*ΔR^2^* = .010, *F*(1,714) = 10.18, *p* = .001). Adding the change in coping using sex in model four did not significantly improve the overall model (*ΔR^2^* = .002, *F*(1,713) = 2.06, *p* = .151).

Based on the most parsimonious model (model 3), being male and living alone predicted lower levels of stress, whilst having a psychiatric condition, being more lonely and greater use of sex to cope prior to lockdown predicted greater levels of stress during lockdown.

Table S6: Results of multiple linear regression on stress during lockdown

| Predictor | Estimate | *SE* | *t* | *p* |
| --- | --- | --- | --- | --- |
| Model 1 |  |  |  |  |
| Gender: |  |  |  |  |
| male – female | -1.027 | 0.363 | -2.832 | 0.005** |
| Age | -0.082 | 0.018 | -4.592 | < .001*** |
| Ethnicity: |  |  |  |  |
| white – not white | 1.076 | 0.422 | 2.551 | 0.011* |
| Education |  |  |  |  |
| degree and above – lower than degree | 0.069 | 0.352 | 0.196 | 0.845 |
| Living Status: |  |  |  |  |
| alone – not alone | -1.255 | 0.554 | -2.266 | 0.024* |
| Previous Diagnosis of Psychiatric Condition: |  |  |  |  |
| yes – no | 2.757 | 0.366 | 7.533 | < .001*** |
| BMI | 0.030 | 0.029 | 1.042 | 0.298 |
| COVID-19 High Risk Health Group: |  |  |  |  |
| high risk condition – no high-risk condition | -0.587 | 0.441 | -1.332 | 0.183 |
| Model 2 |  |  |  |  |
| Gender: |  |  |  |  |
| male – female | -1.036 | 0.330 | -3.139 | 0.002** |
| Age | -0.043 | 0.016 | -2.585 | 0.010** |
| Ethnicity: |  |  |  |  |
| white – not white | 0.800 | 0.383 | 2.086 | 0.037 |
| Education: |  |  |  |  |
| degree and above – lower than degree | 0.215 | 0.320 | 0.674 | 0.501 |
| Living Status: |  |  |  |  |
| alone – not alone | -1.378 | 0.503 | -2.739 | 0.006** |
| Previous Diagnosis of Psychiatric Condition: |  |  |  |  |
| yes – no | 1.587 | 0.345 | 4.600 | < .001*** |
| BMI | 0.011 | 0.026 | 0.417 | 0.677 |
| COVID-19 High Risk Health Group: |  |  |  |  |
| high risk condition – no high-risk condition | -0.361 | 0.400 | -0.902 | 0.367 |
| Loneliness | 0.144 | 0.012 | 12.435 | < .001*** |
| Social Distancing | -0.086 | 0.271 | -0.318 | 0.751 |
| Model 3 |  |  |  |  |
| Gender: |  |  |  |  |
| male – female | -1.492 | 0.358 | -4.172 | < .001*** |
| Age | -0.029 | 0.017 | -1.684 | 0.093 |
| Ethnicity: |  |  |  |  |
| white – not white | 0.670 | 0.383 | 1.748 | 0.081 |
| Education: |  |  |  |  |
| degree and above – lower than degree | 0.183 | 0.318 | 0.575 | 0.566 |
| Living Status: |  |  |  |  |
| alone – not alone | -1.374 | 0.500 | -2.749 | 0.006** |
| Previous Diagnosis of Psychiatric Condition: |  |  |  |  |
| yes – no | 1.622 | 0.343 | 4.727 | < .001*** |
| BMI | 0.005 | 0.026 | 0.193 | 0.847 |
| COVID-19 High Risk Health Group: |  |  |  |  |
| high risk condition – no high-risk condition | -0.306 | 0.398 | -0.769 | 0.442 |
| Loneliness | 0.142 | 0.012 | 12.330 | < .001*** |
| Social Distancing | -0.021 | 0.270 | -0.079 | 0.937 |
| Coping Using Sex Prior to Lockdown | 0.115 | 0.036 | 3.191 | 0.001** |

*Indicates a significance level < .050

**Indicates a significance level < .010

***Indicates a significance level < .001

In an exploratory analysis, we replaced the addition of change in coping using sex in the final step, with the 2-way interaction of gender with coping using sex prior to lockdown. This step did significantly improve the overall model (*ΔR^2^* = .005, *F*(1,714) = 0.745, *p* = .030), such that greater coping using sex prior to lockdown was associated with a greater reported level of stress for females compared to males. For females, coping using sex was significantly associated with levels of stress (β = 0.178, *p* < .001), such that greater coping using sex prior to lockdown predicted greater levels of stress during lockdown. For males, coping using sex was not significantly associated with levels of stress during lockdown (β < -0.001, *p* = .998).
